# Concurrent Antibiotic Therapy in Disseminated Strongyloidiasis

**DOI:** 10.4269/ajtmh.20-1059a

**Published:** 2020-11

**Authors:** Chia Siang Kow, Syed Shahzad Hasan

**Affiliations:** School of Postgraduate Studies; International Medical University; Kuala Lumpur, Malaysia; E-mail: chiasiang_93@hotmail.com; Department of Pharmacy; University of Huddersfield; Huddersfield, United Kingdom; School of Biomedical Sciences & Pharmacy University of Newcastle Callaghan, Australia; E-mail: s.hasan@hud.ac.uk.

Dear Editor,

We read with great interest the case report presented by Lier et al.^1^ on disseminated strongyloidiasis in a patient with COVID-19. This publication serves as a strong reminder for clinicians involved in the management of COVID-19 to consider *Strongyloides* hyperinfection in patients with COVID-19 who demonstrate little or no improvement after receiving medications associated with immunosuppression, especially corticosteroids and tumor necrosis factor inhibitors. In fact, the patient described in this case report received three courses of methylprednisolone, which is a risk factor for developing *Strongyloides* hyperinfection/disseminated disease. Hyperinfection syndrome has been described regardless of dose, duration, or route of corticosteroid administration; even short courses of corticosteroids can predispose patients to the development of hyperinfection/disseminated disease.^2^

Although the patient described in this case report was appropriately initiated on ivermectin (200 mcg/kg/day) when disseminated strongyloidiasis was deemed likely, we feel that the patient should not have had his antibiotic therapy discontinued upon initiation of ivermectin. Rather, antibiotic therapy with activity against enteric Gram-negative bacteria should have been continued. This is significant because part of the life cycle of *Strongyloides stercoralis* involves autoinfection, when the filariform larvae of *S. stercoralis* penetrate the intestinal mucosa (internal autoinfection) or the perianal skin (external autoinfection) to complete the life cycle by migrating via the bloodstream and lymphatics to the lungs and then the intestine.^3^ The penetration of the intestinal mucosa by the filariform larvae of *S. stercoralis* during autoinfection may facilitate entry of enteric Gram-negative organisms into the systemic circulation. Clinically, patients may develop an extraintestinal bacterial infection such as pneumonia, meningitis, or sepsis.

As reported by the authors, the patient demonstrated a lack of significant clinical response to ivermectin and was then suspected to develop pneumonia and eventually meningitis based on the signs and symptoms. It was not until 3 days after the initiation of ivermectin that antibiotic therapy was resumed for suspected pneumonia. This has made us wonder whether, if antibiotic therapy was not discontinued on initiation of ivermectin, the patient may not have developed such a complicated course. The 2018 clinical practice guidelines on the management of strongyloidiasis from the World Gastroenterology Organisation recommend the use of broad-spectrum antibiotics to prevent bacterial meningitis in patients with hyperinfection/disseminated disease.^4^ Among patients who develop hyperinfection/disseminated infection, the mortality rate can be as high as 100%, justifying an aggressive treatment approach in this patient population.^5^
